# Prebiotic Oligosaccharides Potentiate Host Protective Responses against *L. Monocytogenes* Infection

**DOI:** 10.3390/pathogens6040068

**Published:** 2017-12-19

**Authors:** Poyin Chen, Taylor Reiter, Bihua Huang, Nguyet Kong, Bart C. Weimer

**Affiliations:** Department of Population Health and Reproduction, School of Veterinary Medicine, University of California Davis, Davis, CA 95616, USA; pychen@ucdavis.edu (P.C.); tereiter@ucdavis.edu (T.R.); bcahuang@ucdavis.edu (B.H.); nugdao@ucdavis.edu (N.K.)

**Keywords:** prebiotic oligosaccharide, human milk oligosaccharide, eIF2 signaling, unfolded protein response, ER stress, autophagy, cell-mediated immunity (CMI), c-di-AMP

## Abstract

Prebiotic oligosaccharides are used to modulate enteric pathogens and reduce pathogen shedding. The interactions with prebiotics that alter *Listeria monocytogenes* infection are not yet clearly delineated. *L. monocytogenes* cellular invasion requires a concerted manipulation of host epithelial cell membrane receptors to initiate internalization and infection often via receptor glycosylation. Bacterial interactions with host glycans are intimately involved in modulating cellular responses through signaling cascades at the membrane and in intracellular compartments. Characterizing the mechanisms underpinning these modulations is essential for predictive use of dietary prebiotics to diminish pathogen association. We demonstrated that human milk oligosaccharide (HMO) pretreatment of colonic epithelial cells (Caco-2) led to a 50% decrease in *Listeria* association, while Biomos pretreatment increased host association by 150%. *L. monocytogenes*-induced gene expression changes due to oligosaccharide pretreatment revealed global alterations in host signaling pathways that resulted in differential subcellular localization of *L. monocytogenes* during early infection. Ultimately, HMO pretreatment led to bacterial clearance in Caco-2 cells via induction of the unfolded protein response and eIF2 signaling, while Biomos pretreatment resulted in the induction of host autophagy and *L. monocytogenes* vacuolar escape earlier in the infection progression. This study demonstrates the capacity of prebiotic oligosaccharides to minimize infection through induction of host-intrinsic protective responses.

## 1. Introduction

*Listeria monocytogenes* is an intracellular, foodborne, enteric pathogen that is pervasive in livestock, the food supply and the environment. The most at risk populations include children, pregnant women and the elderly. Infection begins with febrile gastroenteritis followed by progression to septicemia, meningitis, or death in immunocompromised individuals and neonatal meningitis or loss of the fetus in pregnant women [1]. Among the major foodborne pathogen-related illnesses, listeriosis presents with the highest mortality rate of any foodborne pathogens [2]. Upon invasion of the intestinal epithelial cell layer, *L. monocytogenes* reside in an intracellular vacuole, followed by vacuolar escape where the organism spreads to neighboring cells parallel to the basal lateral membrane [3]. Left unchecked, infection leads to cell death via apoptosis [4,5].

The molecular basis of *Listeria* pathogenesis is well understood with the identification of E-cadherin as the main receptor for host cell entry and actin comet tails as the mechanism of cell-to-cell dissemination [3]. Transcription profiling during host infection followed by functional characterization reveals novel gene networks controlled by PrfA, lineage-specific transcriptional differences and metabolic network responses to bactericidal treatments [6,7,8]. During *Listeria* infection, listeriolysin O (LLO)-mediated vacuolar escape leads to the host induction of the unfolded protein response (UPR), repression of eIF2 signaling and subsequent endoplasmic reticulum (ER) stress [9,10] in the epithelial cell.

In an effort to minimize the costly gastrointestinal infections and antibiotic abuse, oligosaccharide-based prebiotics are widely used for livestock and human consumption to prevent gastrointestinal pathogen infections [11]. Prebiotic oligosaccharides, such as yeast-derived mannan-oligosaccharides (Biomos) and plant-derived inulin, are composed of repeating chains of a single species of simple carbohydrate monomers connected by a limited repertoire of glycan linkages [12] that are not readily digested by humans. Unlike plant or yeast-derived oligosaccharides, human milk oligosaccharides (HMO) are structurally diverse and comprised of various carbohydrates joined by a variety of chemical linkages [13]. HMO comprise 1–2% of human breast milk composition and is claimed to selectively promote *Bifidobacterium* growth while minimizing pathogen association to neonates [14,15,16]. While not digestible by mammals, prebiotic oligosaccharides can be utilized by and interact with, bacteria in the gut that possess glycosyl hydrolases to digest these compounds such that species-specific interactions and cross feeding occurs between members of the microbiome [17,18]. Prebiotic oligosaccharides are thought to work by acting as host glycan receptor decoys to prevent pathogen association or by binding to glycan receptors on the host cell to alter the host membrane composition, thereby physically impeding pathogen entry [19,20,21]. Conclusive evidence is lacking to demonstrate the specific mechanism to alter food pathogenic bacteria association.

Despite the importance of glycans in cell-to-cell signaling, the role of glycans in gastrointestinal host-pathogen interactions remains a burgeoning area of research due to the complex interplay between microbiota-driven glycan metabolism and host cell signaling [22]. While glycosylation of lipid and wall teichoic acids mediates *L. monocytogenes* virulence, little is known about the role of host and dietary glycans in preventing *L. monocytogenes* infection or in altering infection progression [23,24]. Glycosylation of soluble bioactive molecules can reduce pathogen shedding and direct bacterial host association beyond that of the foundational protein [25,26,27]. Despite identification of E-cadherin as a primary host receptor in *L. monocytogenes* internalization, the effect of E-cadherin glycosylation on *L. monocytogenes* internalization remains unknown [28,29]. Given the diversity of glycans encountered by gut pathogens, we hypothesized that prebiotic oligosaccharides altered *Listeria* host association via modulation of host signaling pathways [30].

Prebiotics are commonly used to modify the intestinal microbiome composition but little is known regarding the effect of prebiotics during infection with an enteric pathogen, such as *L. monocytogenes*. As a whole, bacteria will preferentially metabolize free, fermentable sugars before complex carbohydrates, such as prebiotic oligosaccharides, likely allowing prebiotics to reach the colon in a similar form to which they were consumed. This raises the possibility that prebiotics may to encounter the host epithelium prior to pathogen infection. This guided the experimental design of this study to examine the effect of intact oligosaccharides at pharmaceutically-relevant concentrations on *L. monocytogenes* infection by pre-treating the host prior to infection. This approach provided the basis to test several hypotheses that examined specifics of the molecular mechanisms of prebiotic blocking to reduce pathogen infection, prebiotic-specific host changes and the explicit interaction between prebiotics and *Listeria* to manipulate the host response.

In this study, we characterized the effect of host pretreatment with prebiotics on *Listeria* infectivity as well as the effects of HMO and mannanoligosaccharides on host (Caco-2) and *L. monocytogenes* gene expression during infection of intestinal epithelial monolayers. This led to identification of the prebiotic mechanism of action against *L. monocytogenes* host association that was unexpectedly different between the two oligosaccharides. *Listeria* infection alone induces host stress responses, such as the unfolded protein response (UPR) and endoplasmic reticulum (ER) stress. Only HMO treatment reduced *L. monocytogenes* infection in vitro. *Listeria* infection with HMO pretreatment led to a protective induction of eIF2 signaling, the UPR and subsequent bacterial clearance. In contrast, *Listeria* infection with Biomos pretreatment led to the repression of protective host responses with a concurrent induction of host autophagy.

## 2. Results

### 2.1. HMO Pretreatment Reduces L. monocytogenes Host Invasion

**Figure 1:**
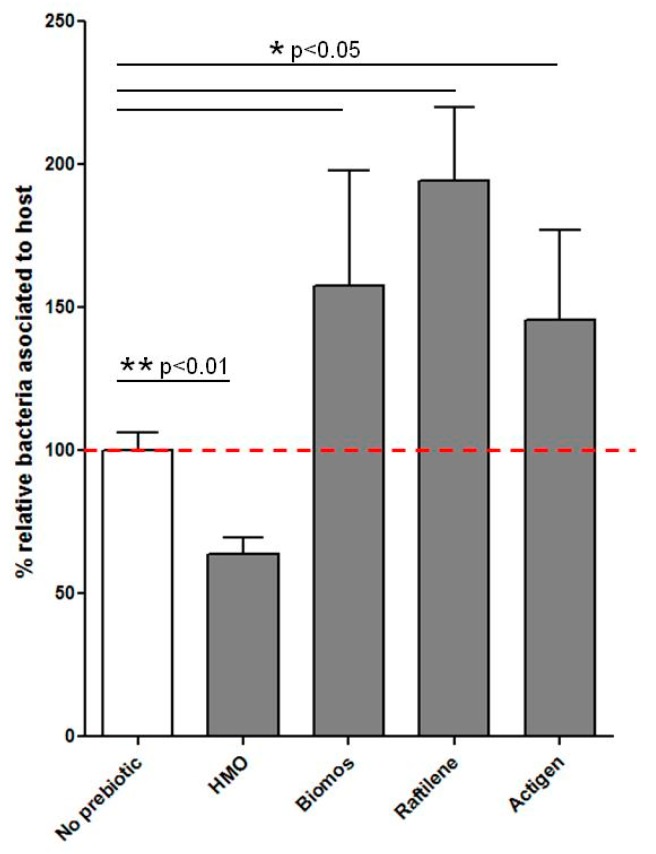
Effect of prebiotic blocking on *L. monocytogenes* association with colonic epithelial cells.

Pathogen blocking capabilities of mannanoligosaccharides (Actigen and Biomos), inulin (Raftilene) and HMO (UC Davis) were tested in vitro during *L. monocytogenes* infection of differentiated Caco-2 monolayers. Of the four prebiotics examined, HMO was the only prebiotic that significantly (*p* < 0.05) decreased *L. monocytogenes* in vitro host association. Pretreatment of Caco-2 monolayers with the mannanoligosaccharides and inulin resulted in a 150% increase in *Listeria* host association compared to the untreated control (Figure 1). Based on these observations, we hypothesized that oligosaccharides have differential effects on the bacterial interactions with the host so as to reduce the bacterial load at the epithelial membrane. To address this, we visualized the impact of initial differential interactions on intracellular progression of *L. monocytogenes* infection.

### 2.2. Prebiotics Modify L. monocytogenes Infection Dynamics

**Figure 2:**
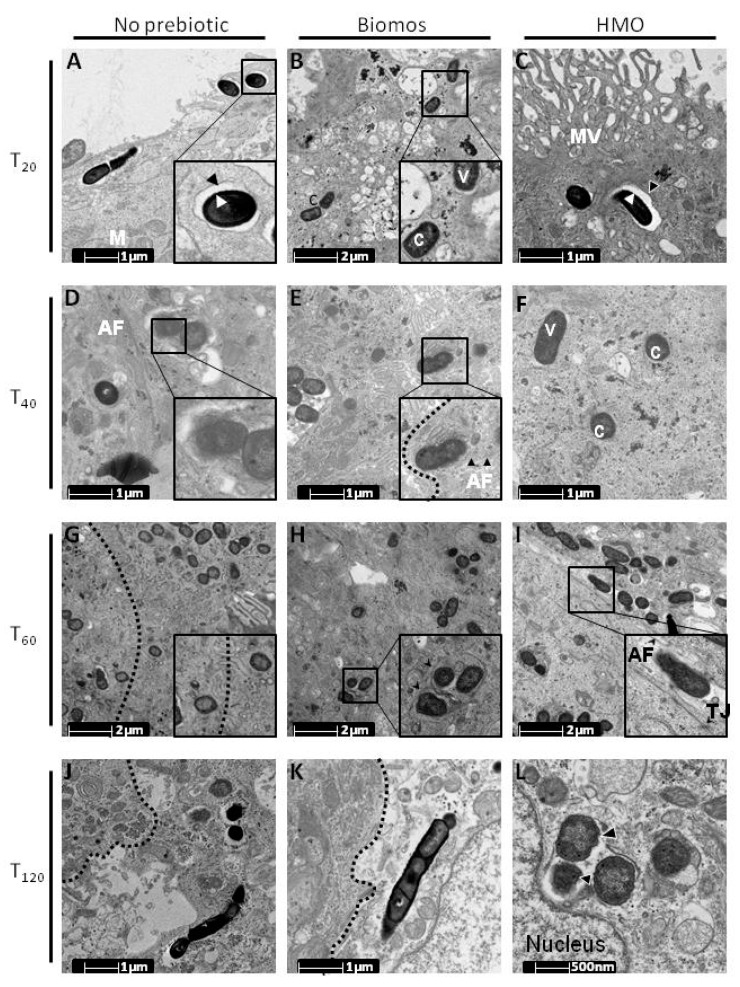
TEM time course of *L. monocytogenes* infection. An infection time course of *L. monocytogenes* with Caco-2 monolayers following 1% HMO and Biomos pretreatment was done. Cytosolic *Listeria* are denoted by “c,” vacuole associated *Listeria* are denoted by “v.” Mitochondria are labeled as “M”; microvilli are labeled as “MV,” actin filaments are labeled as “AF,” tight junctions are labeled as “TJ.” Borders between two cells are indicated by the curved, dotted lines. (**A**–**C**) Intracellular *L. monocytogenes* at 20 min post infection (p.i.). Black and white arrows indicate bacterial and vacuole membrane. (**D**–**F**) *L. monocytogenes* at 40 min p.i. Inserts indicate lateral *L. monocytogenes* infection of neighboring cells along with corresponding actin tails. (**G**–**I**) *L. monocytogenes* at 60 min p.i. Inserts indicate differential infection stages between oligosaccharide pretreatments. (**J**–**L**) *L. monocytogenes* at 120 min p.i. Differential host outcome between oligosaccharide pretreatments. Black arrows indicate *L. monocytogenes* cell membrane ruffling during bacterial clearance by the host.

Due to the observed decrease in host association (Figure 1), we hypothesized that HMO reduced host association via physical blocking mechanism. To test this hypothesis, we initially visualized infected cells with transmission electron microscopy (TEM) using a time course during the infection process. Neither treatment reduced the bacterial association at the membrane but rather changed bacterial localization during the experiment. Biomos-treated cells displayed vacuolar escape within 20 min post infection (p.i.), rather than reduced association. Conversely, *Listeria* infected untreated Caco-2 cells and HMO pretreated cells resulted in vacuoles containing *Listeria* near the apical side of infected host cells after 20 min (Figure 2A–C). At 40 min p.i., we observed *L. monocytogenes* to laterally infect neighboring cells in Biomos pretreated cells. At the same time point, untreated controls and HMO-pretreated samples displayed vacuolar escape and cytoplasmic *L. monocytogenes* (Figure 2D–F).

The effects of prebiotic blocking at 1% concentration on the ability of a pathogen to associate with cultured intestinal epithelial cells following 60 min of infection. No prebiotic control was normalized to 100% and samples were scaled to the control. Assays were carried out in biological and technical replicates (*n* = 3). The mean and SEM of each bar was graphed using Prism5 (GraphPad Software, La Jolla, CA, USA). Statistical calculations were done using JMP (SAS Institute, Raleigh, NC, USA), to calculate/determine the statistical significance between samples as indicated by horizontal bars. * *p* ≤ 0.05; ** *p* ≤ 0.01.

Where initial infection dynamics of HMO pretreatment were similar to the control, as the infection time progressed, infection dynamics of Biomos pretreatment resembled that of the untreated control beginning at 60 min p.i. (Figure 2G–I) with host cell lysis occurring at 120 min p.i. (Figure 2J–K). Bacterial clearance via lysosomes was observed in HMO pretreatment at this time point p.i. (Figure 2).

These observations are counter to the theory that prebiotics act as a blocking molecule. Rather these results expand the possible mechanism of action to include host modification leading to subsequent alteration of host responses to pathogen invasion. We hypothesized a prebiotic-dependent differential host response to be responsible for the observed decrease in *Listeria* host association and thereby, examined host gene expression changes in the context of oligosaccharide pretreatment to elucidate the host mechanisms that are responsible for the observed *Listeria* infection dynamics.

### 2.3. Biomos Drives Differential Expression of L. monocytogenes Genes during Infection

Only one *L. monocytogenes* gene (*lmo_0758*) was significantly differentially regulated (log_2_FC = 0.245, FDR < 0.1) during the HMO treatment. While this gene has not been characterized in *Listeria*, it is homologous to a glyoxalase protein found in *Enterococcus sp*. (100% query coverage, 48% identity, E-value 6e-92) that catalyzes the first step of the methylglyoxalase pathway. This gene is induced under conditions of stress including bile acid and nutrient imbalances [31]. The lack of differential central metabolism expression suggests that *L. monocytogenes* did not widely interact with HMO during the time and constraints of this experiment, counter to proposed models for bacterial modulation for HMO.

In contrast, the presence of Biomos during *L. monocytogenes* infection resulted in repression of 239 bacterial genes (Supplementary Table S2) and surprisingly no induced genes were observed. Of the genes with known function, gene ontology functional characterization revealed repression of numerous catabolism, membrane transport, motility and stress response genes. Gene set enrichment analysis (GSEA) identified numerous genes significantly (*p* < 0.05) repressed that are components of metabolic pathways involved in redox control, amino acid metabolism and secondary metabolite catabolism (Table 1). Of these pathways, vitamin B12 (adenosylcobalamin) metabolism was the most completely reconstructed (*cobD* log_2_FC = −10.807, *p* = 0.000002, FDR = 0.00007; *lmo1164* log_2_FC = −1.858, *p* = 0.0056, FDR = 0.023; *lmo1181* log_2_FC = −1.403, *p* = 0.0129, FDR = 0.0412). Interestingly, three glycosyl hydrolases known to digest mannose-containing glycans were significantly repressed in the presence of Biomos (*lmo0688* log_2_FC = −1.792, *p* = 0.0013, FDR = 0.0095; *lmo0864* log_2_FC = −1.925, *p* = 0.0046, FDR = 0.0207; *lmo2521* log_2_FC = −2.089, *p* = 0.0014, FDR = 0.0095) (Supplementary Table S2). Genes that code for simple sugar PTS transport were repressed that effectively eliminated transport and phosphorylation of glucose, mannose, cellobiose and fructose; thereby blocking their entry into central metabolism (Supplementary Figure S1). Taken together, the repression of catabolic pathways and mannose-targeted glycosyl hydrolases associated with sugar metabolism suggest that not only does the presence of Biomos alter *L. monocytogenes* metabolism but it also influences *L. monocytogenes* cell wall composition, which may explain alteration of the pathogen’s host association capabilities. This observation has not previously been described and may explain why *Listeria* association increased 150% in the presence of Biomos.

## 3. Differential Gene Set Enrichment of Host Cell Signaling Pathways

**Figure 3:**
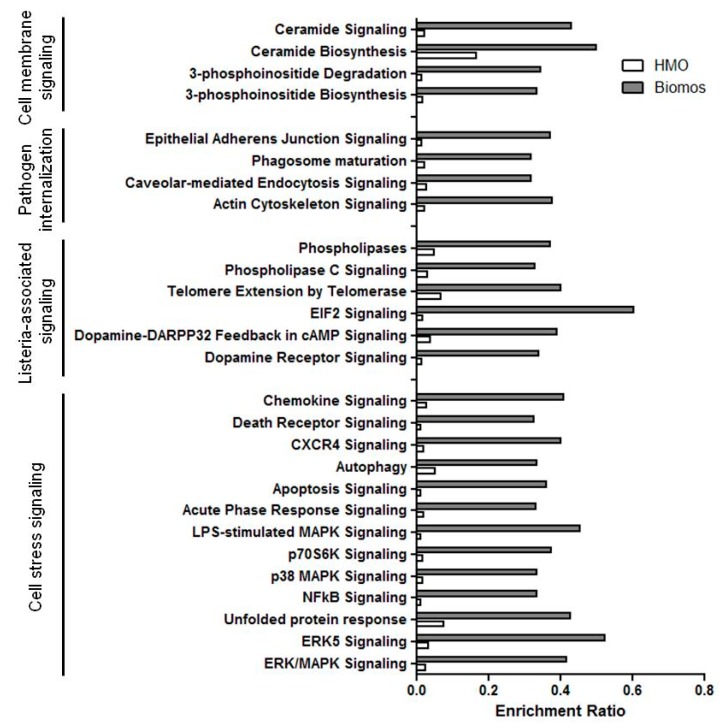
Prebiotic oligosaccharide-dependent differential canonical pathway enrichment following 60 min *L. monocytogenes* infection.

In consideration of the differential localization of *Listeria* and vacuolar escape time between the prebiotic treatments, we hypothesized that prebiotics independently modify the host cell to induce novel signaling pathways that are protective against infection. This experiment resulted in 7752 and 316 significantly (false discovery rate (FDR) < 0.05) differentially expressed genes with Biomos and HMO pretreatment in the colonic cell line, respectively. These genes represented canonical signaling pathways involved with pathogen internalization, regulation of inflammation and cell death. GSEA of host expression revealed a greater number of enriched genes in all pathways when pretreated with Biomos as compared to HMO pretreatment with subsequent *Listeria* infection (Figure 3). These observations support the hypothesis that prebiotics activate intrinsic protective responses in the colonic cell to modulate the progression of *L. monocytogenes* infection. To verify this observation, we examined infection before and after treatment of the host cell with prebiotics.

Ratios due to 1% HMO pretreatment are indicated by the white bars and ratios due to 1% Biomos pretreatment are indicated by the grey bars. Signaling pathways are further classified by functional families, as displayed on the left panel. Enrichment ratios were calculated based on IPA canonical pathways as number of differentially expressed genes over total number of genes in pathway. Ratios were graphed Prism5 (GraphPad Software). 

Examination of genes exhibiting the greatest differential expression revealed modulation of numerous intracellular signaling events that spans from the cell membrane to the nucleus. The presence of HMO during infection resulted in the significant (FDR < 0.05), differential repression of 11 genes, all of which were small RNAs, with the largest repressed gene being SNORA53 with a log_2_ fold change (FC) beyond −2 (Table 2). Conversely, the presence of Biomos during infection resulted in significant (FDR < 0.05) differential expression of 3067 genes that included transcriptional regulators, repression of cell cycle regulation, inflammation and ER stress genes as well as the induction of various transmembrane receptors (Table 2).

We next examined the effect of *L. monocytogenes* infection in the presence of HMO and in the presence of Biomos. Infection in the presence of HMO (HMO/+Lm) resulted in induction of expression while infection in the presence of Biomos (Biomos/+Lm) led to repression of expression (Table 3). The top four canonical pathways and functions that were differentially expressed included ER stress, cell cycle regulation, transcription regulators and small RNAs. These results indicate that prebiotic oligosaccharides were capable of influencing pathogen infectivity via the modulation of host cell signaling pathways that would influence *Listeria* infection, which was observed in this study (Figure 1). Furthermore, the differential activation or repression of host pathways were oligosaccharide-dependent; suggesting that pretreatment with different oligosaccharides lead to varying infection outcomes, as previously observed by modulation with different glycosylated proteins [25,26,27,32].

## 4. Prebiotic-Dependent Differential Expression of Host Cell Stress Signaling during Infection

HMO pretreatment prior to *L. monocytogenes* infection (HMO/+Lm) activated host transmembrane receptors while Biomos pretreatment (Biomos/+Lm) repressed these same receptors (Supplementary Table S3). Twenty-five transcription factors were significantly (*p ≤* 0.0025, FDR ≤ 0.0459) differentially expressed in both Biomos/+Lm and HMO/+Lm. Ten of these transcription factors, including FOS, JUNB and PSMD10, were induced in HMO/+Lm and involved in the host cell cycle regulation and stress response. These same genes were repressed in the Biomos/+Lm treatment (Supplementary Table S3).

Infection with HMO pretreatment resulted in the differential expression of 316 host genes. Only 22 of these genes were unique to this treatment when compared to infection with Biomos pretreatment. Seven out of 22 of these genes were involved in membrane structure and intracellular signaling. Members of the phospholipid signaling pathway involved in host defense against pathogens, LCAT (log_2_FC = −1.17, *p* = 0.00043, FDR = 0.043) and PLA2G3 (log_2_FC = −1.58, *p* = 0.00004, FDR = 0.0171) were significantly repressed. Interestingly, an anti-apoptotic factor-encoding gene, MTRNR2L8, was significantly induced (log_2_FC = 1.41, *p* = 0.00003, FDR = 0.0153) (Supplementary Table S4). Twenty-four cytokines were uniquely expressed during infection with Biomos pretreatment with all but three cytokines being differentially induced (Supplementary Table S4). Taken together, the induction of an anti-apoptotic factor with HMO versus induction of a cytokine-mediated inflammatory response with Biomos, suggests a prebiotic-driven alteration of differential host cell stress and death signaling pathways during *L. monocytogenes* infection rather than a blocking mechanism to alter *Listeria* association. Additionally, these observations support the observed lack of host cell death in this study during infection with HMO treatment in contrast to the host lysis and increase of *Listeria* invasion with Biomos treatment. The observed HMO-induced host survival coupled with the 50% decrease in *L. monocytogenes* host association suggests that HMO pretreatment activates host mechanisms of intracellular pathogen clearance, preventing host death during the time of this experiment.

## 5. Biomos Expedites *L. monocytogenes* Vacuolar Escape

**Figure 4:**
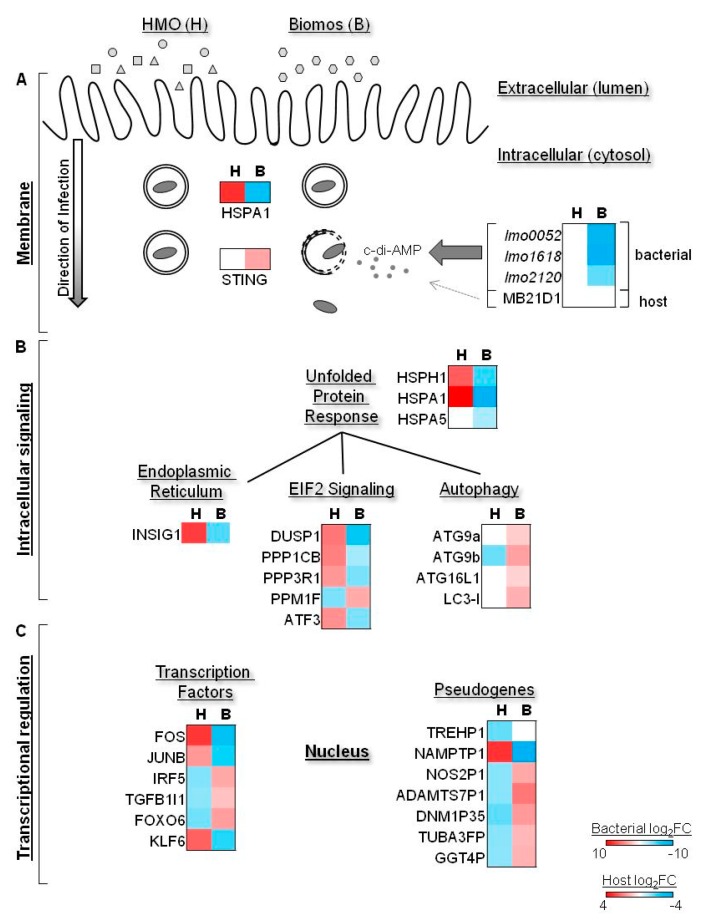
Differential canonical pathway expression during infection in the presence of prebiotic oligosaccharides. Differential expression (log_2_FC) of individual genes visualized by heat map. Bacterial differential expression ranges from −10 < log_2_FC < 10; host differential expression ranges from −4 < log_2_FC < 4. HMO/+Lm is indicated as “H”; Biomos/+Lm is indicated as “B.” HMO is indicated by squares, triangles and circles above the membrane; Biomos is indicated by the hexagons above the membrane. (**A**) Differential subcellular localization is indicated by the intact double circle (H, vacuole associated) versus the dotted line double circle (B, vacuolar escape). c-di-AMP synthesis, transport and degradation genes are listed with arrow weights indicating host vs. bacterial contribution to cytosolic c-di-AMP. (**B**) Differential expression of signaling pathways with prebiotic treatment. (**C**) Differential expression of transcription factors and pseudogenes with prebiotic treatment.

Upon host entry, *L. monocytogenes* is contained within a vacuole that is subsequently lysed by the *L. monocytogenes* virulence effector protein, LLO, to allow vacuole escape and cellular spread during infection progress. Vacuole escape and subsequent access to the cytosol allows *L. monocytogenes* activation of cytosolic host receptors, such as STING, a host receptor for c-di-AMP and an indicator of *L. monocytogenes* vacuole escape. Due to the observation of STING induction in the presence of Biomos, we examined host and bacterial expression of c-di-AMP synthesis and transporter genes to ascertain the source of STING activation. No significant differential expression was observed with host c-di-AMP synthase (MB21D1) with either prebiotic treatment, indicating that there was no prebiotic-driven differential c-di-AMP synthesis by the host. In contrast, *L. monocytogenes* c-di-AMP synthase (*dacA*, lmo2120) was significantly repressed in the presence of Biomos (log_2_FC = −1.75). Interestingly, bacterial c-di-AMP degradation (*pdeA*, lmo0052) and transport (*marR*, lmo1618) were also repressed in the presence of Biomos (log_2_FC = −3.74, log_2_FC = −10.01, respectively) (Supplementary Table S2), which are consistent with the increase in infection. No significant differential expression was observed in these genes with HMO pretreatment. These results demonstrate that Biomos pretreatment decreased *L. monocytogenes* degradation of c-di-AMP, as well as increased transport of bacterial c-di-AMP into the host cytosol. Taken together, these results indicate that *L. monocytogenes* degradation of c-di-AMP was repressed while c-di-AMP transport out of the cell was induced with Biomos pretreatment, indicating that the observed STING induction (Figure 4A) was due to the presence of cytosolic *L. monocytogenes*. To ascertain the consequences of differential *L. monocytogenes* subcellular localization on the host, we next evaluated the differential expression of host signaling pathways involved in responses to *L. monocytogenes* invasion.

## 6. HMO Pretreatment Potentiates Host Cellular Intrinsic Protective Response during *L. monocytogenes* Infection

Given the observed prebiotic-dependent regulation of host signaling pathways (Table 2 and Table 3) coupled with differential *L. monocytogenes* association and subcellular localization (Figure 2), we anticipated drastically different host stress responses to pathogen invasion with different prebiotic treatments. Despite the greater magnitude of host association with Biomos pretreatment, *L. monocytogenes* infection with Biomos pretreatment repressed host protective responses of misfolded protein accumulation with repression of heat shock proteins (HSPH1, HSPA1, HSPA) in the UPR pathway and ER-associated INSIG1. In conjunction with repressed UPR expression, infection with Biomos decreased expression of the ER stress pathway while no change was observed with HMO (Supplementary Figure S1). Infection with Biomos pretreatment also repressed expression of the eIF2 signaling pathway including PPP1CB and numerous phosphatases, as well as stress and inflammatory response transcription factors including ATF3, FOS and JUN. These results are contradictory to the induction of UPR and ER stress that is typically observed with *L. monocytogenes* infection.

Conversely, infection with HMO pretreatment led to induction of UPR and the eIF2 pathway as well as induction of inflammation-associated transcription factors FOS, JUN, IRF5 and TGFB1. Host autophagy, an endpoint to cellular stress and injury, was induced during infection with Biomos pretreatment but repressed with HMO pretreatment (Figure 4B,C). Taken together, these results demonstrate that contrary to the wide-spread belief that prebiotics block pathogens via physical exclusion, addition of prebiotics prior to infection led to modulation of differential pathway activation of many host signal transduction pathways in colonic cells that alter pathogen infection route and duration. While HMO reduced the total *Listeria* association, both prebiotics led to cellular responses to interrupt *Listeria* infection via different mechanisms. Infection in the presence of HMO induced an intrinsic response in epithelial cells to enhance pathogen clearance, while infection in the presence of Biomos potentiated autophagic host cell death.

## 7. Discussion

Successful application of prebiotics, particularly within susceptible populations, such as neonates and immunocompromised individuals, is thought to alter the course of infection from septicemia to mild gastroenteritis. This de-escalation of infection is thought to be accomplished through prebiotic-driven functional enrichment for community members, such as bifidobacteria and lactobacilli. These selected members are capable of metabolizing complex oligosaccharides beyond the capabilities of enteric pathogens; thereby, giving probiotic bacteria a selective advantage to restrict the microbiome community structure to exclude pathogens [17,33,34]. While prebiotic-beneficial microbiota interactions in disease prevention are partially understood, the direct mechanisms of action in prebiotic reduction of pathogens are yet to be described beyond immune-activating capabilities [35]. Inadequate explanations for various microbiome associations leave large gaps in strategic use of oligosaccharides to direct the microbiome to specific endpoint to reduce infection and pathogen carriage [25,26,27].

Prebiotic oligosaccharides are used in agriculture and in the human diet to improve colon health and minimize gut-associated infectious disease. Some studies demonstrate HMO to modulate pathogen association in vitro [16,36]. Despite these observations of pathogen reduction, the impact of prebiotic oligosaccharides on host intracellular protective mechanisms of action, beyond short chain fatty acid production, remains largely unstudied [37]. Cell membrane-associated glycans and protein glycosylation patterns drive cell-to-cell and intracellular signaling in eukaryotic systems [38,39]. Given the structural similarities between prebiotic oligosaccharides and membrane-associated glycans, we sought to elucidate the effect of dietary glycans on *L. monocytogenes* association with the colonic epithelial barrier with the hypothesis that prebiotic oligosaccharides modulated *L. monocytogenes* host association by changing the host membrane and signaling cascades. Mannanoligosaccharides are used to reduce enteric pathogens and shedding in livestock; although, the effect on *L. monocytogenes* infection remains to be explored [16,40].

## 8. Effect of Prebiotics on *L. monocytogenes* Host Association and Gene Expression

Consistent with observations from previous studies using various enteric pathogens, HMO treatment of intestinal epithelial monolayers prior to infection led to a 50% decrease the number of host-associated *L. monocytogenes* (Figure 1) [16]. Interestingly, pretreating Caco-2 monolayers with mannan- and fructo-oligosaccharides increased *L. monocytogenes* infectivity. Studies on the efficacy of mannanoligosaccharides to lower enteric pathogens reported reductions in *Clostridium* and *Escherichia coli*; however, in this study we observed a 150% increase in *L. monocytogenes* infection in the presence of Biomos (Figure 1). Our observations agreed with Park et al. [41], who reported increased *Salmonella* internalization with high mannose glycans. Alterations to the glycosylation profile of host glycoproteins such as the human milk lactoferrin are also associated with modulation of pathogen association with colonic epithelial cells and reduced pathogen shedding [26]. Findings from this study suggest that *L. monocytogenes* recognizes but does not degrade the terminal mannose units of Biomos as a host receptor. Instead of bacterial clearance, this recognition leads to increased pathogen internalization (Figure 1). Due to the differential host association observed with prebiotic treatment, we sought to elucidate *L. monocytogenes* responses to oligosaccharide presence.

The presence of HMO exhibited no transcriptional effect on intracellular *L. monocytogenes*, suggesting that the reduced infective phenotype with HMO pretreatment was a result of HMO acting on host cell receptors and signaling cascades (Supplementary Table S4). In contrast, the presence of Biomos resulted in a repression of many *L. monocytogenes* metabolic pathways, enzymes, replication and cell wall associated genes (Supplementary Table S2)—namely redox, amino acid metabolism and energy metabolism (Table 1). The observed catabolic repression could be attributed to the abundance of mannose-containing glycans in the infection milieu: after 60 min of exposure to high nutrient conditions, the microbe has saturated the input end of carbohydrate metabolic pathways, alleviating the need for continued reaction progression [42]. While one might expect to see an induction of glycosyl hydrolases, the observed expression repression in this study may be a result of the abundance of extracellular mannosyl-oligosaccharides due to Biomos pretreatment, thereby repressing expression of many of these enzymes. Lipoteichoic acid composition has been shown to be necessary for *Listeria* host association and infection [43]. Interestingly, *lmo2521* (*N*-acetylglucosaminyldiphosphoundecaprenol *N*-acetyl-beta-d-mannosaminyltransferase) encodes a glycosyl hydrolase and plays a role in *L. monocytogenes* teichoic acid biosynthesis. While little is known about *Listeria* glycosyl hydrolases, their involvement in shaping teichoic acid composition raises interesting questions about the capacity for prebiotic oligosaccharides to initiate cell wall restructuring in bacteria and their subsequent capacity to interact with the host during infection but suggests that this may play a role in colonic cell association.

Prebiotic oligosaccharides can be metabolized by the intestinal microbiota into molecules, such as short chain fatty acids, that subsequently modulate host cellular responses [37]. The Biomos-induced *L. monocytogenes* metabolic repression observed in this study suggests that in the presence of mannanosyl-oligosaccharides, *L. monocytogenes* metabolism is dampened, leading to decreased *Listeria* metabolism and increased host contact with intact prebiotics.

Vitamin B_12_ (adenosylcobalamin) metabolic genes are induced during *L. monocytogenes* intracellular replication and anaerobic respiration. *L. monocytogenes* synthesis of vitamin B_12_ is thought to generate a pathogenic niche during infection by facilitating growth on ethanolamine as a carbon or nitrogen source to aid in progression of the infection [44]. Our findings of repressed vitamin B_12_ synthesis suggest that Biomos pretreatment either negatively impacts *L. monocytogenes* replication or that *L. monocytogenes* is encountering a less anaerobic environment, relieving the need for vitamin B_12_ to undergo anaerobic respiration with ethanolamine [44]. This is consistent with the observed repression of genes associated with flagellar production, indicating an alternative association mechanism is at work with Biomos and *Listeria*. Given the observation of both bacterial replication at T_60_ with Biomos treatment and host autophagy (Figure 2K), it is likely that the repression of vitamin B_12_ metabolic genes was due to an aerobic environment brought about by host cell death.

## 9. Prebiotic-Driven *L. monocytogenes* Vacuolar Escape

**Figure 5:**
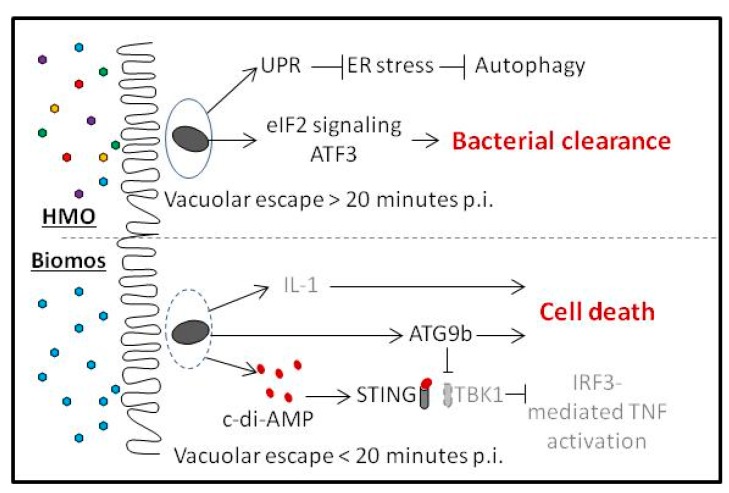
Model of prebiotic-dependent differential host gene expression following *L. monocytogenes* infection.

Upon host internalization, *L. monocytogenes* initially resides in a vacuole prior to accessing the cytosol. Vacuolar escape and subsequent cytosolic access is enabled by expression of the bacterial virulence factor, listeriolysin O [45]. Different phases of *L. monocytogenes* infection are benchmarked by induction of specific host genes: heat shock protein 70 (HSP70) as marker for early infection, vacuole-associated *L. monocytogenes* and stimulator of interferon genes (STING) as a marker for cytosolic *L. monocytogenes* [46,47]. Hsp70 plays a dual role during infection: stabilization of the phagosome to prevent vacuolar escape and as a chaperone for misfolded proteins [48]. Although HSP70 induction is typically associated with early (15 min p.i.) *L. monocytogenes* infection, we found HSP70 to be induced (Figure 4A) with cytosolic *L. monocytogenes* by 60 min p.i. (Figure 2I), suggesting a protective role for Hsp70 against *L. monocytogenes*-induced misfolded proteins [48]. Upon vacuolar escape, cytosolic *L. monocytogenes* secretes c-di-AMP, activating the host c-di-AMP binding protein STING [49]. Differential repression of *L. monocytogenes* c-di-AMP degradation (lmo0052) and a c-di-AMP transporter repressor (lmo1618) coupled with repression of host cGAMP synthase (MB21D1) point to *L. monocytogenes* c-di-AMP as the source of STING induction (Figure 4A) [50]. Furthermore, HSP70 induction by HMO treatment opposed to STING induction with Biomos suggests prebiotic-dependent differential subcellular localization of intracellular *L. monocytogenes* (Figure 2A–C,M, Figure 4A and Figure 5). Time course TEM imaging of *L. monocytogenes* infection revealed enhanced *L. monocytogenes* vacuolar escape with Biomos pretreatment prior to 20 min p.i. while *L. monocytogenes* in control and HMO-treated samples remained vacuole-associated (Figure 2A–C). Although eventual vacuolar escape and lateral infection is observed in HMO treated cells (Figure 2C,F,I), bacterial clearance by the host at 60 min p.i. is unique to HMO pre-treatment (Figure 2M). In contrast, infected host cells in both control and Biomos-treated samples were observed to undergo cell death (Figure 2J,K).

## 10. Host Cell Intrinsic Responses against *L. monocytogenes* Invasion

Early vacuolar escape into the cytosol provides an opportunity for *Listeria* to damage the colonic cell using various methods that broadly disrupt signal transduction that ultimately end in cell death. Gene expression modification of oligosaccharide-treated Caco-2 monolayers during infection (HMO/+Lm and Biomos/+Lm) revealed a global reduction in pathogen-induced and host stress signaling pathway enrichment with HMO, showing that infection in the presence of Biomos modulated 40-fold more genes than did infection in the presence of HMO (Figure 3). Despite the canonical pathway enrichment disparity between prebiotic treatments, analysis of differentially expressed genes shared between both treatments revealed changes in key genes involved in the regulation of cell cycle, inflammation, ER stress and cell structure (Table 2). Additionally, expression profiles were inversely related between HMO/+Lm and Biomos/+Lm—genes induced with one treatment were repressed with the other treatment. As expected, the addition of extrinsic glycans in the form of prebiotic oligosaccharides modulated host membrane receptors during *L. monocytogenes* infection (Supplementary Table S3). Our observation of differentially expressed inflammatory mediators and differentiation-related transcriptional regulators suggests direct modulation of intracellular transcriptional responses within the host (Supplementary Table S3). In addition to previously reported effects of prebiotic oligosaccharides on immunomodulation, our results indicate changes to host differentiation patterns, which have only been reported in mesenchymal stem cells infected with bacteria [51]. These unexpected results indicate that prebiotics are capable of directly reshaping the transcriptomic landscape of epithelial cells to prime an antimicrobial response in non-phagocytic host cells.

Biomos pretreatment directly and negatively regulated inflammation and ER stress but induced expression of cell surface receptors and cell structure components (Table 2). Although HMO itself did not result in any observable changes in expression of signaling pathways at 60 min p.i. (Table 2), the identification of differentially regulated small RNAs suggests ability for HMO to prime cellular immunity, allowing for a more rapid response if infection should occur [52]. This HMO-driven differential regulation of transcription factors has been previously observed in cases of allergic and infection-driven immune activation [53,54]. Unexpectedly, numerous small RNAs were found to be significantly and highly modulated with prebiotic treatment. This observation is in line with findings from previous transcriptome profiling studies [55,56,57]. Additionally, our findings suggest that prebiotic oligosaccharides are not only able to modulate the host cell surface but they are capable of influencing host post-transcriptional modification systems. Small nucleolar RNA (SNORA53) was the only RNA-coding gene to be differentially expressed in Lm/+HMO but not in Lm/+Biomos (Table 2). Although the target sequences for SNORA53 have yet to be mapped, this finding suggests that the presence of HMO during *L. monocytogenes* infection initiates post-transcriptional modification of a different subset of mRNA compared to Biomos [58,59]. Broadly, these results suggest that transcriptome modulation pattern is specific to different species of prebiotic oligosaccharides. The role in the infection biology remains unclear, in spite of the large regulatory changes with HMO.

Lecithin-cholesterol acyltransferase (LCAT) and phospholipase A2 group III (PLA2G3) were two genes uniquely regulated by HMO/+Lm that were not differentially regulated in the Biomos/+Lm treatment (Supplementary Table S4). LCAT and PLA2G3 are both involved in the phospholipid signaling pathway. Changes in LCAT abundance can result in modulation of host membrane fluidity and cholesterol concentration while PLA2G3 is a member of secreted phospholipase A2 family with protective activity against microbial pathogens [60,61,62]. The finding of these two uniquely differentially expressed genes in HMO/+Lm provides additional evidence of HMO-induced alterations to the colonic cell surface and subsequent signaling cascades. These alterations contribute to a host response of active reduction *L. monocytogenes* association rather than a passive response by the prebiotic in blocking pathogen adhesion.

STING, a host marker for cytosolic c-di-AMP, was induced during *L. monocytogenes* infection with Biomos pretreatment. We determined *L. monocytogenes* to be the source of c-di-AMP during Biomos treatment due to the observed repression of *Listeria* c-di-AMP degradation and transport repressor, with a concurrent lack of differential host c-di-AMP synthesis (Figure 4). Despite the observed STING induction, no significant differential expression of the genes involved in a subsequent interferon regulatory factor 3 (IRF3)-dependent type 1 interferon (IFN) response were observed. This is likely due to repression of the STING binding partner, tank binding kinase 1 (TBK1) (Figure 4B and Figure 5), whose induction is needed to activate IRF3 [63].

The activation of the autophagy related 9b (ATG9b) gene, an apoptosis enucleation factor and a negative regulator of TBK1, supports the observation of TBK1 repression and corroborates the observed induction of the apoptosis signaling pathway with Biomos pretreatment [64]. These results suggest that while *L. monocytogenes* host invasion with Biomos pretreatment follows the canonical steps of initial host entry, the presence of Biomos during infection represses the subsequent host cell intrinsic protective response [47,49,65]. This repression ultimately results in increased bacterial burden and increased host death.

Oligosaccharide pretreatment is indicated by the colored hexagons with Biomos indicated by blue hexagons and HMO indicated by multicolored hexagons. Vacuole-associated *L. monocytogenes* are depicted by dark grey ovals (*Listeria*). Enhanced vacuolar escape with Biomos pretreatment is indicated by dotted grey line.

*L. monocytogenes* host invasion modulates numerous signal transduction pathways including eukaryotic initiation factor 2 (eIF2) signaling and the unfolded protein response (UPR) [9,10]. eIF2 signaling serves to modulate mRNA translation in response to stress while UPR acts to contain and control damages made by stress-induced protein misfolding. As a part of eIF2 signaling, induction of activating transcription factor 3 (ATF3) during intracellular bacterial infection enhances pathogen clearing [66]. Consistent with previous findings, induction of the eIF2 pathway and ATF3 expression with HMO pretreatment was correlated with decreased intracellular *L. monocytogenes* burden (Figure 1 and Figure 4B,C) as well as with enhanced pathogen clearing and decreased host cell death at 120 min post infection with HMO pretreatment (Figure 2 and Figure 5) [9,66]. In contrast, repression of the UPR with Biomos pretreatment likely allowed for a buildup of infection-induced misfolded cytosolic proteins [10]. This occurrence contributed to the induction of apoptosis signaling and subsequent increased host cell death (Figure 3 and Figure 4B), allowing for pathogen dissemination to begin another round of infection into neighboring host cells [65].

## 11. Materials and Methods

### 11.1. Oligosaccharides

Raftiline was obtained from Orafti (Lot# HPBNL23JP3, Malvern, PA, USA). Bio-BIOMOS and Actigen were supplied by Alltech Inc. (Nicholasville, KY, USA). HMO was isolated and gifted to the Weimer lab by Dr. Daniela Barile (UC Davis, CA, USA) [67]. Plant and yeast-derived oligosaccharides were in powder form and stored dry at room temperature. HMO was stored at −20 °C until use. All oligosaccharides were resuspended in high glucose DMEM (HyClone Laboratories, Logan, UT, USA) at a 1% working concentration.

### 11.2. Bacterial Strains and Growth Conditions

*L. monocytogenes* strain EGD-e was obtained from ATCC (BAA-679). Stock cultures were stored in nonfat dry milk with glycerol at −80 °C. *L. monocytogenes* cultures were grown in brain heart infusion (BHI) broth. Cultures were incubated in BHI broth for 4 to 6 h at 37 °C to reach exponential phase or for 14 h to reach early stationary phase. Second transfers were carried out by transferring 1% of the exponential phase cultures to fresh BHI broth and incubated at 37 °C for 14 h.

### 11.3. Human Cell Line and Growth Conditions

Human colonic carcinoma (Caco-2) cell lines were obtained from ATCC (HTB-37). Caco-2 stocks were stored in DMEM with 10% DMSO in liquid nitrogen. Thawed Caco-2 cells were grown in DMEM with 10 mM MOPS (Sigma, St. Louis, MO, USA), 10 mM TES (Sigma, St. Louis, MO, USA), 15 mM HEPES (Sigma, St. Louis, MO, USA), 2 mM NaH_2_PO_4_ (Sigma, St. Louis, MO, USA), 20% fetal bovine serum (HyClone Laboratories), 1% glutamax (Thermo Scientific, Rockford, IL, USA), 1% PenStrep (Thermo Scientific, Rockford, IL, USA) and 1% non-essential amino acids (Thermo Scientific, Rockford, IL, USA). Cells were grown to confluency and differentiated for 12 to 15 days before use in gentamicin protection assays. Caco-2 cells were seeded into 96-well plates 14 days prior at 10,000 cells/cm^2^. Culture medium was renewed every three days.

### 11.4. In Vitro Colonic Cell Infection Assays

Colonic cell (Caco-2) infection assays were performed as previously described [68] and modified by Shah et al. [69,70]. Briefly, differentiated Caco-2 cells were pre-treated with 1% oligosaccharide suspended in serum-free DMEM. Following a 15-min incubation in the presence of oligosaccharides, Caco-2 cells were exposed to early stationary phase *L. monocytogenes* (*n* = 3; multiplicity of infection = 1000) for 60 min. Cells were washed with PBS buffer (pH = 7.2) and lysed with 50 ml Warnex buffer (AES Chemunex Canada, Inc., Montreal, QC, Canada) following manufacturer’s directions.

Warnex lysis was deactivated by incubation at 95 °C for 15 min. Samples were diluted 1:10 in nuclease-free water and stored at −20 °C pending qPCR quantification as described by Arabyan et al. using forward (F) and reverse (R) PCR primers (F) 5′-CTCAAATACGAATGCTAACCAAGGT-3′ and (R) 5′-TTTGAGCTTCAGCAATAATAGCACTT-3′ [22]. Quantification was done using qPCR with results reported as number of *L. monocytogenes* colony forming units (cfu)/Caco-2 cell. All samples were assayed in biological and technical triplicate and graphed using the statistical software, GraphPad InStat 3 (GraphPad Software, Inc., La Jolla, CA, USA) and ANOVA with Tukey’s test using JMP version 10 (SAS Institute, Triangle Park, NC, USA). Data are displayed as the mean ± SEM with three biological replicates. *p* < 0.05 was considered to be statistically significant.

### 11.5. RNA Extraction

Differential host lysis was done with infected samples by passaging cells through a 22 gauge needle [71]. Bacterial cells were enzymatically lysed following the protocol used by the 100 K Pathogen Genome PROJECT for bacterial lysis [72]. Samples were pelleted via centrifugation and suspended in Trizol LS. RNA was extracted from TRIzol LS (Ambion #10296) following manufacturer instructions. RNA purity (A_260/230_ and A_260/280_ ratios ≥ 1.8, ≤ 2.0) and integrity were confirmed with Nanodrop (Nanodrop Technologies, Wilmington, DE, USA) and BioAnalyzer RNA kit (Agilent Technologies Inc., Santa Clara, CA, USA).

### 11.6. RNAseq Library Preparation

Double-stranded cDNA was synthesized from purified RNA using the SuperScript Double-Stranded cDNA Synthesis kit (11917-010; Invitrogen, Carlsbad, CA, USA) following manufacturer’s instructions. cDNA quality was assayed using the NanoDrop 2000 spectrophotometer (Nanodrop Technologies) and the Agilent 2100 BioAnalyzer (Agilent Technologies Inc.) [73]. Sequencing libraries were constructed from 100ng of double-stranded cDNA from each sample following using the KAPA HyperPlus library preparation kit (kk814, KAPA Biosystems, Boston, MA, USA) with adaptors from BIOO Scientific NEXTFlex (514105, BIOO, Austin, TX, USA). Quality control for fragment size distribution was done using the High Sensitivity kit (Agilent Technologies Inc.) while library concentration was measured using the KAPA SYBR FAST qPCR kit Master Mix (2x) Universal (kk4903; KAPA) on Bio-Rad CFX96 (Bio-Rad). Libraries were indexed at eight libraries per lane and sequenced with PE150 on a HighSeq4000 at the California Institute for Quantitative Biosciences in the Vincent J Coates Genomics Sequencing Lab (Berkeley, CA, USA).

### 11.7. Statistical Analysis for Differential Gene Expression

Prior to gene expression analysis the raw sequence reads were trimmed using Trimmomatic [74] and aligned to the Ensembl GRCh38 human genome using HISAT2 [75] using an index downloaded from the HISAT2 website on 11/22/16 (grch38_tran). Reads were aligned in paired-end mode with soft clippings permitted. Paired-end reads that did not map to the human genome were separated and subsequently aligned to the *Listeria monocytogenes* genome (GCA_000196035.1_ASM19603v1; European Nucleotide Archive sample accession SAMEA3138329) using Bowtie2 [76]. All alignment files output by HISAT2 and Bowtie2 were compressed using samtools [77]. This file was used for differential expression analysis.

Gene counts were estimated using featureCounts in the Rsubread R package [78] and differential gene expression analysis was performed in edgeR [79]. Gene counts were produced using the Ensembl GRCh38.86.gtf annotation for human and the GCA_000196035.1_ASM19603v1.gtf annotation file for *L. monocytogenes*. The gene count tables produced by featureCounts were separately entered in to edgeR where normalization and differential expression were performed. Genes with counts per million less than one and with expression in fewer than two samples per group were discarded. Reads were normalized using the library size. A design matrix was implemented based on treatment. Because there were three or four biological replicates in each group, dispersion was estimated for each treatment. Treatment groups contained pairwise comparisons so the edgeR exact test was used for differential expression estimation. Significance was defined as adjusted *p*-value (FDR, this was done using a Bonferroni correction) of less than or equal to 0.05.

No reads aligned to the *L. monocytogenes* genome from uninfected cells; therefore, no differential expression analyses were performed for *L. monocytogenes* from these samples; differential expression was performed only between *L. monocytogenes* infected samples.

Raw reads are publically available on the NCBI Sequence Read Archive (SRA). SRA identification codes for each sample are available in Supplementary Table S1. Data and codes used for differential expression analyses are available on Github (https://github.com/bartweimer/Listeria_prebiotic_oligosaccharides).

### 11.8. Gene Expression Pathway Analysis

Host differential expression visualization and biological pathways were done using Ingenuity Pathway Analysis software (IPA, Ingenuity Systems, QIAGEN, Redwood City, CA, USA) [80]. Significantly different signaling pathways were determined using IPA with a two-tailed Fisher’s exact test (*p ≤* 0.05) and canonical pathway enrichment.

Visualization of *L. monocytogenes* differential expression data was performed using BioCyc SmartTables (Pathway Tools version 21.0, SRI International, Menlo Park). Gene set enrichment analysis was done using a two-tailed Fisher’s exact test (*p* ≤ 0.05) with the Pathway Tools *L. monocytogenes* strain EGD-e database version 21.0 [81] as described by Rivals et al. [82].

### 11.9. Transmission Electron Microscopy

TEM was performed at the UC Davis Electron Microscopy Core as previously described [73]. Cells were grown on Lab-Tek^®^ Permanox Chamber 8-Well Slides from Electron Microscopy Sciences, P.O. Box 550, 1560 Industry Road, Hatfield, PA, USA. Culture media was removed and at least 100 μL of modified Karnovsky’s fixative (2.5% glutaraldehyde and 2.0% paraformaldehyde in 0.1 M phosphate buffer, pH 7.3) was added per well and fixed for a minimum of 1 h. The cells were washed in 0.1 M phosphate buffer and secondarily fixed for 1 hour in freshly made 1% osmium tetroxide and 1.5% potassium ferrocyanide in ddH_2_0. Fixative was washed three times from the cells with cold ddH_2_O. Dehydration follows through ascending concentrations of ethanol (30%, 50%, 70%, 95%, 100%) with a minimum of 10 min in each and three changes of 100%. A 100% concentration of epon-araldite resin lacking nadic methyl anhydride was added to each well and allowed to infiltrate overnight at room temperature. The next day as much resin as possible was removed from each well. New resin was added to fill the wells completely. Slides were polymerized at 70 °C. Once polymerized, area of interest was chosen and cut to block size with a fine bladed (jewelers) saw. Sections were cut with a Leica UCT ultramicrotome (Leica Ultracut UCT, Leica, Vienna, Austria) and stained with uranyl acetate and lead citrate before viewing in the Talos L120C electron microscope at 80 KV. Images were acquired using the fully integrated Ceta CBIOMOS camera (FEI/TheroBiomoscientific Company, Hillsboro, OR, USA, made in Eindhoven, The Netherlands).

## 12. Conclusions

Studies to define the mechanism of prebiotic efficacy in minimizing infection risk have focused on the secondary metabolites produced by intestinal bacteria and oligosaccharide blocking. In this study, we demonstrated that host pretreatment with HMO led to a 50% decrease in *L. monocytogenes* host association while pretreatment with Biomos led to a 150% increase in host association. Biomos treatment repressed the expression of numerous catabolic pathways and influenced *Listeria* glycosyl hydrolase activity on teichoic acid synthesis whereas HMO did not significantly modulate *L. monocytogenes* gene expression. Host pretreatment with HMO potentiated a protective stress response with induction of UPR and eIF2 signaling, enabling enhanced bacterial clearance by the host and subsequent host survival. In contrast, host pretreatment with Biomos hastened *L. monocytogenes* vacuolar escape as evidenced by STING induction and by TEM and resulted in induction of host autophagy. Altogether, this study revealed an unprecedented ability for prebiotic oligosaccharides to influence host cellular signaling pathways and host and *Listeria* survival outcomes but they were not due to blocking, rather colonic cell changes in the intrinsic response.

## Figures and Tables

**Table 1:** Gene set enrichment analysis of Biomos-induced differentially expressed *L. monocytogenes* genes at 60 min p.i. (Significance changes in enrichment were calculated using a two-tailed Fisher’s exact test with *p* ≤ 0.05).

Function	Pathways	*p*-Value	Genes Matched
Redox	Cobalamin Biosynthesis	0.003	*cobD*, lmo1164, lmo1181
	Cofactors, Prosthetic Groups, Electron Carriers Biosynthesis	0.008	*nadE, nadD, hemE, menE, hemA, cobD, serC, panB, thyA,* lmo2749, lmo2101, lmo0938, lmo1930, lmo1164, lmo1181
	Vitamins Biosynthesis	0.012	*cobD, serC, panB, thyA,* lmo2749, lmo2101, lmo1164, lmo1181
	Adenosylcobalamin Biosynthesis	0.008	lmo1164, lmo1181
	Adenosylcobalamin salvage from cobalamin	0.008	
	Biotin-carboxyl carrier protein assembly	0.022	*accD*, lmo1357
	Vitamin B_6_ Biosynthesis	0.022	*serC*, lmo2101
Amino acid metabolism	Amino Acids Degradation	0.006	*gltD, aspB,* lmo2836, lmo2363, lmo0383, lmo1182, lmo2101, lmo2749, lmo1915
	Proteinogenic Amino Acids Degradation	0.006	
	L-aspartate Degradation	0.022	*aspB*, lmo1915
	Aspartate degradation II	0.022	
	Aspartate superpathway	0.047	*nadE, nadD, aspB*, lmo1436, lmo1437
	Pyrimidine deoxyribonucleosides salvage	0.022	*thyA*, lmo1463
	Pyrimidine deoxyribonucleotides de novo biosynthesis II	0.042	*thyA*, lmo0279
	L-threonine Biosynthesis	0.039	*aspB*, lmo1436, lmo1437
	Threonine biosynthesis	0.039	
Energy metabolism	Degradation/Utilization/Assimilation	0.026	*gltD, aspB*, lmo0347, lmo2696, lmo1057, lmo2648, lmo2836, lmo1463, lmo0877, lmo1182, lmo0383, lmo2101, lmo2749, lmo0372, lmo0917, lmo2771, lmo2840, lmo1915, lmo2362, lmo2095, lmo2835
Secondary metabolite degradation	Taxiphyllin bioactivation	0.028	lmo0372, lmo0917, lmo2771
	Nitrogen Containing Glucosides Degradation	0.028	
	Cyanogenic Glucosides Degradation	0.028	
	Nitrogen Containing Secondary Compounds Degradation	0.039	

**Table 2:** Comparison of the differential gene expression with *Listeria* infection in Lm/+HMO and Lm/+Biomos treatments following 60 min of infection. Genes with no significant differential expression are denoted by “ns” and their corresponding LogCPM, *p*-value and FDR values are denoted by “--.” Significance is defined as FDR ≤ 0.05. Gene IDs are provided where possible. Ensembl IDs of each gene are listed.

Gene Function	Gene Name	Ensembl ID	Lm/+HMO				Lm/+Biomos			
			Log_2_ FC	LogCPM	*p*-Value	FDR	Log_2_ FC	LogCPM	*p*-Value	FDR
RNA-associated proteins, small RNAs and pseudo-genes	SNORA53/ACA53	ENSG00000212443	−3.347	1.672	3.25 × 10^−8^	0.00021	ns	--	--	--
	RPPH1	ENSG00000277209	−1.420	4.499	2.5 × 10^−5^	0.04977	−3.598	4.651	2.5 × 10^−11^	2.8 × 10^−9^
	RPPH1	ENSG00000259001	−1.417	4.502	2.5 × 10^−5^	0.04977	−3.584	4.653	1.9 × 10^−11^	2.3 × 10^−9^
	AC010761.8	ENSG00000264577	−1.302	3.546	7.6 × 10^−7^	0.00374	−3.269	3.650	1.6 × 10^−18^	2.1 × 10^−15^
	RN7SL5P	ENSG00000278249	−1.553	1.421	2.5 × 10^−6^	0.00605	−1.797	1.729	2.3 × 10^−5^	2.0 × 10^−4^
	SCARNA2	ENSG00000270066	−1.553	1.421	2.5 × 10^−6^	0.00605	−1.797	1.729	2.3 × 10^−5^	2.0 × 10^−4^
	RN7SL5P	ENSG00000265735	−1.243	2.999	1.5 × 10^−6^	0.00503	−1.658	3.278	3.0 × 10^−5^	2.4 × 10^−4^
	SnoU2_19	ENSG00000275146	−1.138	1.452	1.2 × 10^−6^	0.00451	−1.340	1.747	1.1 × 10^−3^	4.3 × 10^−3^
	RMRP	ENSG00000277027	−1.316	3.178	2.6 × 10^−8^	0.00021	−1.182	3.614	1.1 × 10^−2^	2.9 × 10^−2^
	RMRP	ENSG00000269900	−1.315	3.178	2.8 × 10^−8^	0.00021	−1.182	3.614	1.1 × 10^−2^	2.9 × 10^−2^
	AC084809.2	ENSG00000226377	ns	--	--	--	−3.743	2.709	5.4 × 10^−20^	9.3 × 10^−17^
	RPL23A	ENSG00000198242	ns	--	--	--	−3.530	6.238	3.2 × 10^−25^	1.4 × 10^−21^
	RP11-3P17.5	ENSG00000269888	ns	--	--	--	−3.511	1.359	3.6 × 10^−21^	8.9 × 10^−18^
	RPL23AP42	ENSG00000234851	ns	--	--	--	−3.511	1.359	3.6 × 10^−21^	8.9 × 10^−18^
	RN7SL2	ENSG00000274012	ns	--	--	--	−3.330	7.607	5.1 × 10^−11^	4.9 × 10^−9^
	AL627171.2	ENSG00000282885	ns	--	--	--	−3.317	7.609	3.2 × 10^−11^	3.4 × 10^−9^
	AC010761.1	ENSG00000264577	ns	--	--	--	−3.269	3.650	1.6 × 10^−18^	2.1 × 10^−15^
	RN7SL255P	ENSG00000239808	ns	--	--	--	−2.975	0.630	5.3 × 10^−8^	1.4 × 10^−6^
	AC121158.1	ENSG00000231335	ns	--	--	--	−2.707	2.933	4.6 × 10^−13^	9.6 × 10^−11^
	MALAT1	ENSG00000278217	ns	--	--	--	−2.701	3.849	2.05 × 10^−13^	4.8 × 10^−11^
	C11orf98	ENSG00000278615	ns	--	--	--	−2.683	1.110	5.45 × 10^−17^	4.5 × 10^−14^
	CCDC74A	ENSG00000163040	ns	--	--	--	2.013	0.254	2.40 × 10^−8^	7.2 × 10^−7^
	AC022966.2	ENSG00000267601	ns	--	--	--	2.057	0.132	3.68 × 10^−7^	6.7 × 10^−6^
	NRA5A1	ENSG00000136931	ns	--	--	--	2.078	2.664	2.93 × 10^−18^	3.5 × 10^−15^
	RP11-432M8.5	ENSG00000249329	ns	--	--	--	2.079	0.360	3.51 × 10^−11^	3.6 × 10^−9^
	AC113208.3	ENSG00000260660	ns	--	--	--	2.111	0.784	5.66 × 10^−9^	2.2 × 10^−9^
	AC017104.6	ENSG00000224376	ns	--	--	--	2.122	−0.211	4.61 × 10^−10^	2.9 × 10^−8^
	KRT16P2	ENSG00000227300	ns	--	--	--	2.142	−0.021	2.7 × 10^−8^	7.8 × 10^−7^
	PPP6R2P1	ENSG00000233442	ns	--	--	--	2.148	−0.062	9.5 × 10^−9^	3.4 × 10^−7^
	CA15P1	ENSG00000241527	ns	--	--	--	2.172	−0.143	4.3 × 10^−6^	5.0 × 10^−5^
	FAM90A22P	ENSG00000215365	ns	--	--	--	2.189	−0.027	1.8 × 10^−9^	8.7 × 10^−8^
	BTBD17	ENSG00000204347	ns	--	--	--	2.215	0.772	1.2 × 10^−9^	6.5 × 10^−8^
	RP11-26H16.4	ENSG00000283234	ns	--	--	--	2.309	0.035	1.6 × 10^−11^	2.0 × 10^−9^
	RP11-331F4.5	ENSG00000280152	ns	--	--	--	2.348	0.207	3.5 × 10^−10^	2.3 × 10^−8^
	SLC25A34	ENSG00000162461	ns	--	--	--	2.904	−0.043	4.9 × 10^−12^	7.5 × 10^−10^
Tran-scriptional regulators	ZBED6	ENSG00000257315	ns	--	--	--	−2.625	2.892	5.7 × 10^−15^	2.6 × 10^−12^
	ZNF703	ENSG00000183779	ns	--	--	--	2.680	2.689	1.3 × 10^−18^	1.9 × 10^−15^
	HO × D10	ENSG00000128710	ns	--	--	--	2.041	0.792	8.3 × 10^−11^	7.4 × 10^−9^
Cell cycle	NAMPTP1	ENSG00000229644	ns	--	--	--	−3.272	0.963	1.5 × 10^−26^	8.6 × 10^−23^
	GADD45A	ENSG00000116717	ns	--	--	--	−2.684	2.171	3.0 × 10^−16^	2.0 × 10^−13^
Inflammation	TNFAIP3	ENSG00000118503	ns	--	--	--	−3.054	4.040	7.2 × 10^−21^	1.6 × 10^−17^
	JUN	ENSG00000177606	ns	--	--	--	−3.436	3.904	3.9 × 10^−33^	4.4 × 10^−29^
	FOS	ENSG00000170345	ns	--	--	--	−2.871	3.398	9.4 × 10^−16^	5.4 × 10^−13^
Endoplasmic reticulum stress	HSPA1A	ENSG00000204389	ns	--	--	--	−2.883	3.702	1.8 × 10^−34^	4.2 × 10^−30^
	HSPA1B	ENSG00000204388	ns	--	--	--	−2.749	3.423	6.5 × 10^−30^	4.9 × 10^−26^
	PSMD10	ENSG00000101843	ns	--	--	--	−2.749	1.833	1.0 × 10^−22^	3.9 × 10^−19^
Cell structure	SPTBN5	ENSG00000137877	ns	--	--	--	2.017	3.286	3.7 × 10^−16^	2.4 × 10^−13^
	MYL3	ENSG00000160808	ns	--	--	--	2.125	2.038	4.5 × 10^−15^	2.1 × 10^−12^
Receptors	GRASP	ENSG00000161835	ns	--	--	--	2.068	1.589	1.1 × 10^−13^	2.8 × 10^−11^
	TMEM82	ENSG00000162460	ns	--	--	--	2.091	0.168	1.6 × 10^−8^	5.1 × 10^−7^
	CHRND	ENSG00000135902	ns	--	--	--	2.130	0.405	1.9 × 10^−10^	1.4 × 10^−8^
	UCHL1	ENSG00000154277	ns	--	--	--	2.165	3.558	7.8 × 10^−19^	1.2 × 10^−15^
	GPR142	ENSG00000257008	ns	--	--	--	2.211	0.751	2.1 × 10^−11^	2.4 × 10^−9^
	GFRA4	ENSG00000125861	ns	--	--	--	2.218	0.069	5.2 × 10^−9^	2.1 × 10^−7^
	CABP7	ENSG00000100314	ns	--	--	--	2.285	1.519	3.1 × 10^−10^	2.1 × 10^−8^

**Table 3:** Comparative transcriptomics of Biomos differentially expressed genes in HMO/+Lm and Biomos/+Lm following 60 min of infection. Genes with no significant differential expression are denoted by “ns” and their corresponding LogCPM, *p*-value and FDR values are denoted by “--.” Significance is defined as FDR ≤ 0.05. Gene IDs are provided where possible. Ensembl IDs of each gene are listed.

Gene Function	Gene Name	Ensembl ID	HMO/+Lm				Biomos/+Lm			
			Log_2_ FC	LogCPM	*p*-Value	FDR	Log_2_ FC	LogCPM	*p*-Value	FDR
ER stress and protein folding	YOD1	ENSG00000180667	1.927	1.527	1.3 × 10^−5^	9.1 × 10^−3^	−2.505	1.413	1.1 × 10^−14^	1.1 × 10^−11^
	HSPA1B	ENSG00000204388	3.146	3.801	1.8 × 10^−9^	1.1 × 10^−5^	−3.009	3.528	6.2 × 10^−30^	3.5 × 10^−26^
	HSPA1A	ENSG00000204389	3.228	4.070	1.1 × 10^−10^	1.2 × 10^−6^	−3.145	3.813	1.1 × 10^−32^	1.6 × 10^−28^
	HSPH1	ENSG00000120694	2.062	4.095	3.3 × 10^−5^	1.5 × 10^−2^	−1.579	3.787	4.6 × 10^−12^	1.6 × 10^−9^
	INSIG1	ENSG00000186480	2.432	4.979	2.6 × 10^−6^	4.3 × 10^−3^	−1.480	4.788	5.7 × 10^−8^	3.2 × 10^−6^
	PSMD10	ENSG00000101843	1.912	1.654	2.6 × 10^−6^	1.3 × 10^−2^	−2.668	1.579	2.6 × 10^−12^	1.0 × 10^−9^
Inflammation	TNFAIP3	ENSG00000118503	ns	--	--	--	−2.897	3.777	1.4 × 10^−32^	1.6 × 10^−28^
	NFKB1A	ENSG00000100906	ns	--	--	--	−2.591	2.723	4.0 × 10^−22^	1.5 × 10^−18^
	JUN	ENSG00000177606	ns	--	--	--	−3.407	3.751	1.7 × 10^−31^	1.3 × 10^−27^
Cell cycle	GADD45A	ENSG00000116717	2.083	2.346	4.0 × 10^−5^	1.7 × 10^−2^	−2.878	2.215	6.8 × 10^−25^	3.1 × 10^−21^
	TXNIP	ENSG00000265972	2.645	5.494	5.5 × 10^−5^	1.8 × 10^−2^	−2.066	5.187	4.4 × 10^−12^	1.5 × 10^−9^
	NAMPT	ENSG00000105835	2.325	5.180	2.3 × 10^−5^	1.2 × 10^−2^	−2.363	5.024	1.3 × 10^−12^	5.8 × 10^−10^
	NAMPTP1	ENSG00000229644	2.563	0.994	6.5 × 10^−6^	7.4 × 10^−3^	−3.402	0.934	5.5 × 10^−22^	1.8 × 10^−18^
Miscellaneous functions	TIPARP	ENSG00000163659	2.557	5.719	3.4 × 10^−6^	5.3 × 10^−3^	−1.055	5.777	1.2 × 10^−5^	1.9 × 10^−4^
	CD164	ENSG00000135535	2.280	5.575	4.8 × 10^−5^	1.7 × 10^−2^	−1.970	5.610	2.7 × 10^−10^	4.5 × 10^−8^
	GGCT	ENSG00000006625	2.154	1.467	1.1 × 10^−5^	9.1 × 10^−3^	−2.651	1.532	2.2 × 10^−10^	3.9 × 10^−8^
Transcriptional regulator	FOS	ENSG00000170345	2.520	3.030	4.6 × 10^−8^	1.5 × 10^−4^	−2.468	2.890	2.4 × 10^−13^	1.4 × 10^−10^
	ZNF703	ENSG00000183779	−2.001	2.473	2.9 × 10^−4^	3.8 × 10^−2^	2.141	2.674	4.6 × 10^−16^	5.7 × 10^−13^
	ZBED6	ENSG00000257315	2.622	2.535	5.6 × 10^−6^	7.4 × 10^−3^	−2.181	2.362	7.9 × 10^−11^	1.7 × 10^−8^
	KLF6	ENSG00000067082	2.010	5.008	1.6 × 10^−4^	3.0 × 10^−2^	−1.625	4.845	8.8 × 10^−8^	4.5 × 10^−6^
RNA⁻associated proteins, small RNAs and pseudogenes	HNRNPA1	ENSG00000135486	2.397	5.602	5.1 × 10^−5^	1.7 × 10^−2^	−2.433	5.567	8.5 × 10^−17^	1.2 × 10^−13^
	RPL23A	ENSG00000198242	2.198	5.435	2.3 × 10^−8^	8.7 × 10^−5^	−3.133	5.742	4.1 × 10^−21^	1.2 × 10^−17^
	RPPH1	ENSG00000277209	1.596	3.351	1.9 × 10^−6^	3.6 × 10^−3^	−2.905	3.885	1.7 × 10^−8^	1.3 × 10^−6^
	MRPL36	ENSG00000171421	2.083	1.352	1.4 × 10^−9^	1.1 × 10^−5^	−2.502	1.916	4.0 × 10^−15^	4.5 × 10^−12^
	RN7SL255P	ENSG00000239808	4.349	0.639	3.2 × 10^−9^	1.5 × 10^−5^	−2.712	0.329	2.2 × 10^−13^	1.4 × 10^−10^
	KRT17P4	ENSG00000205312	−1.575	0.132	4.3 × 10^−4^	4.3 × 10^−2^	2.258	0.067	8.1 × 10^−11^	1.8 × 10^−8^
	RPL23AP42	ENSG00000234851	1.831	0.804	1.3 × 10^−5^	9.1 × 10^−3^	−3.126	0.824	7.1 × 10^−14^	5.2 × 10^−11^
	AC084809.2	ENSG00000226377	4.358	2.636	2.3 × 10^−15^	5.4 × 10^−11^	−3.823	2.643	5.6 × 10^−18^	1.1 × 10^−14^
	AC107072.2	ENSG00000231335	2.105	2.386	4.4 × 10^−5^	1.7 × 10^−2^	−1.749	1.998	4.6 × 10^−11^	1.2 × 10^−8^
	CTD-202417.13	ENSG00000246422	2.286	1.332	6.4 × 10^−6^	7.4 × 10^−3^	−1.227	1.357	1.1 × 10^−6^	3.1 × 10^−5^
	MACC1-AS1	ENSG00000228598	2.466	1.806	6.7 × 10^−7^	1.8 × 10^−3^	−2.101	1.410	5.2 × 10^−13^	2.8 × 10^−10^
	CTD-3014M21.1	ENSG00000279602	2.594	2.937	9.4 × 10^−6^	8.6 × 10^−3^	−1.983	2.744	1.7 × 10^−11^	4.8 × 10^−9^
	RPPH1	ENSG00000259001	1.590	3.357	2.0 × 10^−6^	3.6 × 10^−3^	−2.893	3.890	1.4 × 10^−8^	1.1 × 10^−6^
	RP11-3P17.5	ENSG00000269888	1.823	0.806	1.0 × 10^−5^	8.9 × 10^−3^	−3.126	0.824	7.1 × 10^−14^	5.2 × 10^−11^
	RPS13	ENSG00000110700	1.784	5.309	4.2 × 10^−5^	1.7 × 10^−2^	−2.705	5.665	1.5 × 10^−13^	10.0 × 10^−11^
	AC010761.8	ENSG00000264577	1.296	2.570	3.2 × 10^−4^	3.9 × 10^−2^	−2.607	2.914	6.5 × 10^−13^	3.4 × 10^−10^
	RN7SL2	ENSG00000274012	ns	--	--	--	−2.892	7.104	3.8 × 10^−8^	2.4 × 10^−6^
	RP11-596C23.6	ENSG00000282885	ns	--	--	--	−2.880	7.107	2.6 × 10^−8^	1.8 × 10^−6^
	DNASE1L3	ENSG00000283148	ns	--	--	--	2.226	−0.175	6.2 × 10^−11^	1.5 × 10^−8^
	RP11-321E2.8-001	ENSG00000250055	ns	--	--	--	2.400	−0.013	4.9 × 10^−13^	2.7 × 10^−10^

## Supplementary Materials

The following are available online at www.mdpi.com/2076-0817/6/4/68/s1.
