# Supplementary material for: Prebiotic Oligosaccharides Potentiate Host Protective Responses against L. Monocytogenes Infection
Source: Pathogens. 2017 Dec 19;6(4):68. doi: 10.3390/pathogens6040068 (PMC5750592; doi:10.3390/pathogens6040068)
Supplement: Supplementary file 1 [file pathogens-06-00068-s001.zip › supp data/Supplementary Figure.pdf]

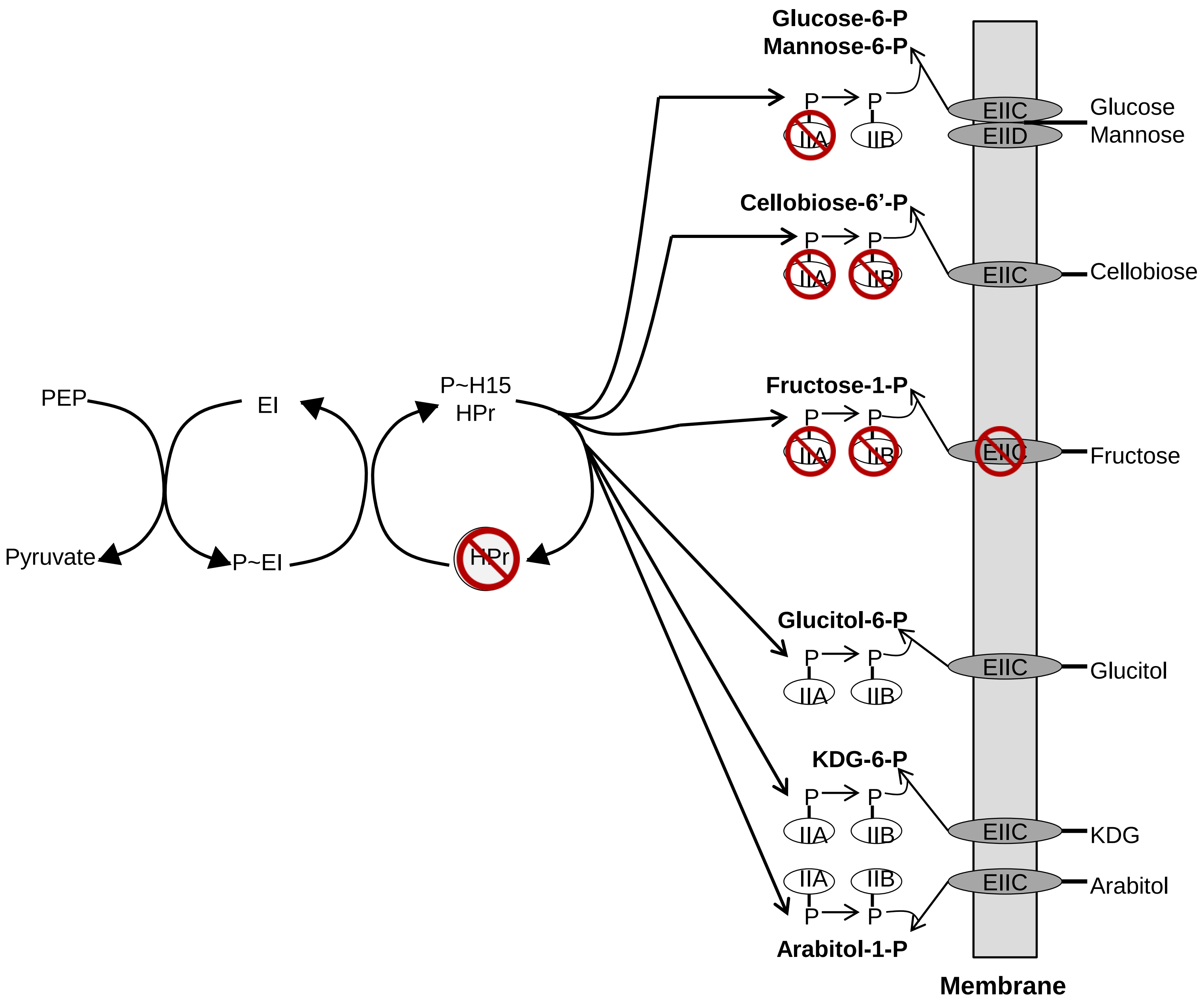

Supplementary Figure 1. Biomos-repressed *L. monocytogenes* sugar PTS and phosphotransferase genes during infection

Effect of infection on cells pretreated with HMO

Effect of infection on cells pretreated with Biomos

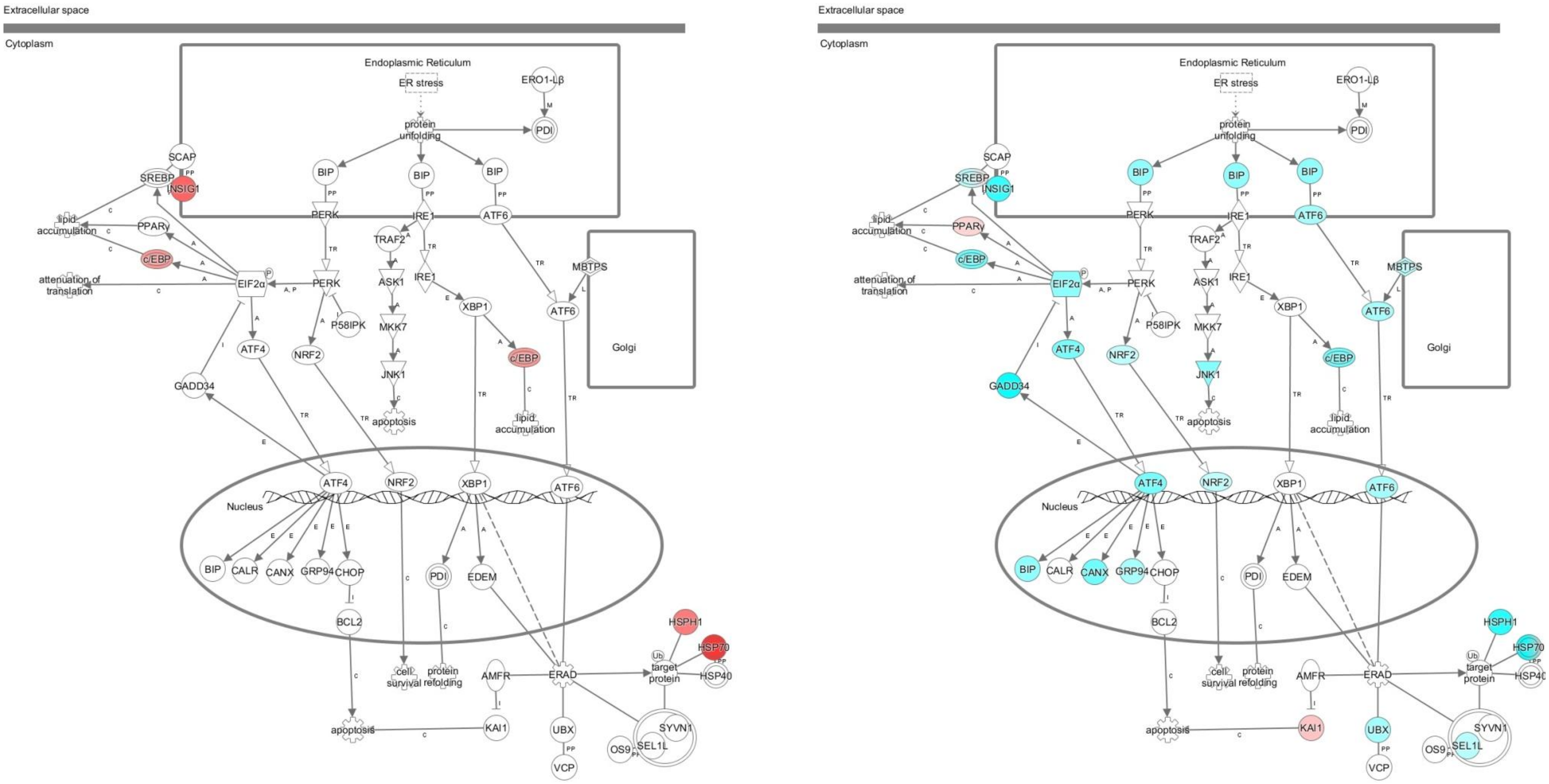

Supplementary Figure 2. Pathogen-induced differential Unfolded Protein Response signaling with oligosaccharide pretreatment.
